# Supplementary material for: Exploring the perceived impact of physical activity on physical and mental health among individuals with long COVID: A qualitative interview inquiry
Source: PLoS One. 2026 May 27;21(5):e0350121. doi: 10.1371/journal.pone.0350121 (PMC13215512; doi:10.1371/journal.pone.0350121)
Supplement: S1 Table — Code descriptions. (DOCX) [file pone.0350121.s001.docx]

Supplementary Table 1. Code descriptions

| Physical Health- Worsened | Description |
| --- | --- |
| Fatigue/exhaustion | Fatigue, exhaustion, or tiredness related to PA. Fatigue or exhaustion not bound within specific time following PA. |
| Crash/feel worse after | Worsening of any long COVID symptoms following PA immediately or within hours of when PA performed. General descriptions of worsening with PA or multiple worsening symptoms coded here. |
| Worse next day | Worsening of long COVID symptoms the day following PA. Any worsening symptoms attributed to PA the day following PA coded here. |
| No energy for PA | Reports feeling too fatigued or having no energy to perform PA. Not able to perform as much PA, including ADLs, following prior PA performance. Cyclical worsening of fatigue with PA coded here. |
| Heart rate (too slow or fast) | Tachycardia or bradycardia attributed to PA. Not present prior to long COVID or worsened with long COVID. |
| Shortness of breath | Shortness of breath attributed to PA. Not present prior to long COVID or worsened with long COVID. |
| Can’t get out of bed | Bedbound following PA. Largely limited to bed following PA performance. Time bedbound varies (hours to weeks). |
| Must take breaks | Requires breaks during PA, including ADLs, that participant did not require prior to long COVID. |
| Dizziness/balance issues | Feeling off balance, dizzy, or lightheaded either during or following PA. Attributed to PA. |
| Temperature regulation issues | Feeling too warm or too cold with inability to properly regulate body temperature. Not present prior to long COVID or worsened with long COVID. Attributed to PA. |
| Chest pain | Chest pain attributed to PA. Not present prior to long COVID or worsened with long COVID. |
| Worsened other conditions | Other health conditions worsen during or following PA. Examples include asthma and chronic pain. Not present prior to long COVID or worsened with long COVID. |
| Undesired changes in weight | Undesired losing weight or gaining weight since long COVID, which the participant attributes to PA. |
| Physical Health- Improved |  |
| “Exercise is good” belief | Reporting education that exercise and PA are good for health, and participant believes exercise likely does or will improve their long COVID symptoms. |
| Feels stronger | Feeling stronger following PA. Notes improvements in muscle tone or strength. |
| Improved in nature | Feeling improved physical symptoms when performing PA in nature. |
| Energized by exercise | Feeling energized and increasing energy during or following PA. |
| Physical Health- Unchanged |  |
| Unchanged by physical activity | Specifically reports feeling physical health does not change with PA. |
| Mental Health- Worsened |  |
| Cannot do what they used to | Loss of a particular activity that the participant was able to perform prior to long COVID. |
| Forced inactive lifestyle | Feeling forced into an inactive lifestyle though the participant previously engaged in or wishes to engage in different lifestyle. |
| Frustration | Reports frustration or irritation by current PA abilities or effects of PA. |
| Cannot do what they want | Unable to perform physical activities or job that is desired due to long COVID. |
| Grief or loss | Grieves or reports loss regarding prior identity that involved PA or athlete identity. |
| Unpredictability of PA effects | Reports effects of PA vary, making it difficult to determine how the body will respond to PA bout. Lack of control of PA effects. |
| Fear/anxiety regarding PA | Reports worries of acute issues with PA, such as a fall, syncope, panic attack, or asthma attack. |
| Sadness | General sadness about PA abilities. Not specifically related to prior identity, as in grief or loss |
| Anger | Reports anger at self or others about PA abilities or effects of PA. Examples include anger regarding inability to perform desired PA or anger at healthcare provider for suggesting unrealistic PA. |
| Trapped | Reports feeling trapped by inability to be active. Often related to being largely stuck indoors. |
| Sensory overload | PA associated with sensory overload, such as light, tactile, or auditory overload. |
| Regret following PA | Reports regretting PA following PA performance. Due to several causes including worsened symptoms following PA, grief, or frustration. |
| Mental Health- Improved |  |
| Mood improved after | Reports general improved mood following PA. |
| Sense of accomplishment | Sense of accomplishment or achievement following PA. |
| Feeling hopeful | Reports feeling hopeful about future with long COVID with PA. Includes noting improved strength and decreased symptoms with PA as well as being able to perform more PA over time. |
| Noting improvements | Specifically notes improvement in ability to perform PA, such as walking for longer or lifting heavier weights. |
| Being outdoors | Reports improved mood with performing PA outdoors. |
| Mental Health- Unchanged |  |
| Unchanged by PA | Specifically reports feeling mental health does not change with PA. |

^a^ PA = physical activity
